# Investigation of New Accelerometer Based on Capacitive Micromachined Ultrasonic Transducer (CMUT) with Ring-Perforation Membrane

**DOI:** 10.3390/mi15020279

**Published:** 2024-02-16

**Authors:** Luhao Gou, Hongliang Wang, Qi Ding, Yulong Liu, Runze Yang, Feng Zhang, Pengcheng Zhang, Gang Cao

**Affiliations:** National Key Laboratory for Electronic Measurement Technology, Key Laboratory of Instrumentation Science & Dynamic Measurement, Ministry of Education, North University of China, Taiyuan 030051, China; s202106043@st.nuc.edu.cn (L.G.); b20220631@st.nuc.edu.cn (Q.D.); sz202106081@st.nuc.edu.cn (Y.L.); s202206134@st.nuc.edu.cn (R.Y.); b20210629@st.nuc.edu.cn (F.Z.); s202106145@st.nuc.edu.cn (P.Z.); s202106099@st.nuc.edu.cn (G.C.)

**Keywords:** CMUT, accelerometer, acceleration sensitivity, ring perforations

## Abstract

Capacitive micromachined ultrasonic transducer (CMUT) has been widely studied due to its excellent resonance characteristics and array integration. This paper presents the first study of the CMUT electrostatic stiffness resonant accelerometer. To improve the sensitivity of the CMUT accelerometer, this paper innovatively proposes the CMUT ring-perforation membrane structure, which effectively improves the acceleration sensitivity by reducing the mechanical stiffness of the elastic membrane. The acceleration sensitivity is 10.9 (Hz/g) in the acceleration range of 0–20 g, which is 100% higher than that of the conventional CMUT structure. This research contributes to the acceleration measurement field of CMUT and can effectively contribute to the breakthrough of vibration acceleration monitoring technology in aerospace, medical equipment, and automotive electronics.

## 1. Introduction

With the increasing demand for miniaturization, low power consumption, and multifunctional sensors, MEMS sensors have become a popular research direction due to the advantages of microelectromechanical system devices, such as their miniaturization, low power consumption, batch fabrication [1], high degree of integration, and ability to measure multiphysical quantities, which can reduce size and mass without sacrificing functionality [2]. Micro-machined accelerometers include several types, such as piezoresistive, capacitive, piezoelectric, and resonant accelerometers. Among many MEMS accelerometers, resonant accelerometers have the advantages of high measurement accuracy, strong anti-interference ability, and better long-term stability than other types of sensors. They have been extensively researched in recent decades [3,4,5], including medical devices, consumer electronics, aerospace, and automotive electronics [2,4,6].

Due to the proven potential of widely applied resonant accelerometers, the rapidly advancing CMUT (capacitive micro-machined ultrasound transducer) sensor, as one of the members of the resonant sensor category, is introduced for the first time in this paper for application in acceleration measurement. CMUT-based accelerometers, based on capacitive micro-machined ultrasound transducer technology, are more conducive to achieving large-scale integration and simultaneous measurement of multiple physical quantities due to their composition of multiple resonant units. Thanks to their multiple resonators, they exhibit higher stability during testing [7] and enhanced resistance to interference. Additionally, CMUT accelerometers, driven by electrostatic forces, offer an output range unrestricted by the power supply voltage [1]. Therefore, CMUT-based accelerometers can emerge as robust candidates for MEMS (microelectromechanical systems) resonant accelerometer systems.

The initial intention of the capacitive microelectromechanical ultrasonic transducer (CMUT) is to achieve a working frequency range in the MHz range. It is widely used as a resonant element for biomedical detection, ultrasound imaging, and viscosity measurement. Based on the significant advantages of CMUT, people have gradually considered expanding its applications to include the detection of pressure, humidity, gas density, etc. The capacitive micromachined ultrasonic transducer (CMUT) can be equated to a parallel plate capacitor [4,8,9,10]. When a DC bias voltage is applied to an elastic plate with fixed edges, the elastic plate deforms, resulting in a change in resonant frequency.

Numerous studies have been conducted to address this phenomenon, such as the capacitive micromachined ultrasonic transducer (CMUT)-based resonant chemosensors proposed in 2011 and 2018 [1,11]; the effect of electrostatic force and uniform hydrostatic pressure on the resonant frequency of edge-fixed circular microplates was investigated in 2013 [12]; a dual-frequency capacitive micromachined ultrasonic transducer (CMUT) pressure transducer capable of detecting two pressure ranges was proposed in 2019 [5]; a biomolecule weight detection sensor based on a capacitive micromachined ultrasonic transducer (CMUT) was proposed in 2014 [13]; a (CMUT) gas sensor for detecting CO_2_ concentration was investigated in 2016 [14]; and a humidity sensor based on a capacitive micromachined ultrasonic transducer (CMUT) was proposed in 2019 [15,16].

With the emergence of more and more capacitive micromachined ultrasonic transducer (CMUT)-based transducers, more extensive research on CMUT-based transducers is needed to realize highly integrated and multifunctional CMUT transducers. Due to the relatively small thickness of the designed membrane structure, the process of etching to form a ring-perforation structure on the silicon membrane presents significant challenges. This paper primarily investigates the CMUT (capacitive micro-machined ultrasound transducer) accelerometer through finite element simulation, utilizing the COMSOL Multiphysics simulation software. The study focuses on an array-type sensor capable of measuring acceleration. Initially, the operational principles of the CMUT accelerometer are theoretically analyzed. Subsequently, a finite element model is established to analyze the frequency response of the CMUT unit under applied loads. Structural optimization is then conducted. To enhance the sensitivity of the CMUT accelerometer, a ring-perforation structure in the membrane is proposed in this study. By creating finite element models with varying perforation numbers, radii, and positions, the impact of the ring-perforation structure on acceleration sensitivity is investigated. Detailed analyses are provided for the number, radius, and position of the ring perforations. The ring-perforation structure aims to increase acceleration sensitivity by reducing the effective thickness of the elastic membrane and consequently lowering its mechanical stiffness. The paper also conducts a comparative analysis between the traditional structure of the CMUT accelerometer and the ring-perforation structure.

## 2. Theoretical Analysis of CMUT Acceleration Sensor

**Figure 1:**
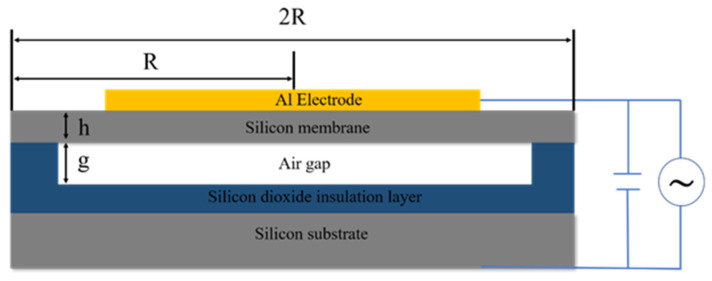
Schematic diagram of parallel plate capacitor under electrostatic force.

This paper designs a CMUT sensor for detecting acceleration. The CMUT single-cell structure is shown in Figure 1. The CMUT cell structure is similar to a flat capacitor structure, consisting of metal electrodes (including top and bottom electrodes), a vibrating membrane, edge support, a cavity, an insulating layer, a substrate, etc. CMUT sensors are composed of a parallel connection of different numbers of cell structures. When a DC and AC voltage is applied to the CMUT device, the elastic membrane of the CMUT vibrates. When an external load (such as acceleration) is applied to the CMUT device, the resonant frequency of the CMUT device changes. The vibrating film of a capacitive micromachined ultrasonic transducer (CMUT) belongs to a circular elastic thin plate [17,18,19], and the elastic stresses generated inside the film cause the strain of the vibrating film and cause it to vibrate after deformation by force. Thin plate vibration is an important problem in engineering practice. In the analysis of the vibration characteristics of elastomers, CMUT elastic film vibration displacement, velocity and natural frequency, and other characteristics can be obtained.

In the free vibration problem of a thin plate, the plate is in equilibrium under a certain transverse load. The plate deviates from the equilibrium position under external disturbing forces (acceleration and pressure). When the external disturbing factors are removed, the plate vibrates with a slight amplitude near the equilibrium position. The fixed electrode and the microplate in the balanced plate capacitor model are equivalent to a pair of conductive electrode plates. When a voltage V is applied between the two plates, the two plates are subjected to a distributed electric field force. The governing equations for the bending of the thin plate are as follows [5,9,20,21]:

(1) $$m\ddot{x}+c\dot{x}+kx=Q\left(t\right)$$

In Equation (1), $Q\left(t\right)$ represents the electrostatic force. $Q\left(t\right)=\frac{\varepsilon AV^{2}\left(t\right)}{2(g-x{)}^{2}}$. According to Equation (1), the following is when the acceleration is zero:

(2) $$\frac{1}{2}\frac{\varepsilon AV^{2}}{(g-x{)}^{2}}=k\cdot x$$

The following is when the acceleration is not equal to zero:

(3) $$\frac{1}{2}\frac{\varepsilon AV^{2}}{(g-x-\Delta x{)}^{2}}=k\left(x-\Delta x\right)-m\cdot a$$

In Equation (1), (x−Δx) represents the displacement of the elastic membrane when the acceleration is not equal to zero.

In Equation (1), k is the mechanical stiffness of the elastic membrane plate, c is its equivalent damping coefficient, and m is the mass of the elastic membrane plate. In the sealed air-cavity CMUT structure, gas introduced between the moving surface and the fixed surface leads to the introduction of membrane damping [22,23,24]. The air between the plates affects the system by increasing its stiffness and damping [25,26]. Therefore, it is necessary to consider the influence of membrane damping. The structure depicted in this paper consists of parallel plates with an inflatable cavity, and the governing differential equations for motion can be expressed as the following [27]:

(4) $$m\ddot{x}+c\dot{x}+(k+k_{S})x=Q\left(t\right)\;$$

Here, $k_{S}$ represents the air spring constant, and as described by Ayrat Galisultanov et al.in this paper [27]. The expression for $k_{S}$ is as follows:

(5) $$K_{s}=\frac{P_{a}A}{g}f_{d}\left(\sigma \right)\;$$

In Equation (5), $P_{a}$ is the ambient pressure, A is the area of a single cell, and g is the height of the cavity. In this approach, we assume the sealed circular plate has no viscous damping [25], meaning $f_{d}\left(\sigma \right)=1$. The intrinsic resonance frequency of the elastic membrane plate when no external load is applied is as follows:

(6) $$f_{0}=\frac{1}{2\pi }\sqrt{\frac{k}{m}}\;$$

The resonant frequency after considering the spring softening due to electrostatic forces and the damping effect of membrane compression is as follows:

(7) $$f_{e}=\frac{1}{2\pi }\sqrt{\frac{k-k_{e}-k_{S}}{m}}$$

In Equation (7), $K_{e}=\frac{\varepsilon AV^{2}(t)}{g^{3}}$. From Equation (7), it can be seen that after the electrostatic force is applied, due to the electrostatic force, the membrane plate produces the ‘soft spring effect’ resulting in the resonance frequency shift. When the acceleration is zero (only the electrostatic load is applied), the resonant frequency of the membrane becomes smaller due to the ‘soft spring effect’ effect. When the acceleration is not zero, and the amplitude of the DC bias is much larger than the amplitude of the AC voltage, the frequency $f_{e}$ of the membrane can be expressed as the following:

(8) $$f_{e}=\frac{1}{2\pi }\sqrt{\frac{k-k_{S}-\frac{\varepsilon AV^{2}}{{\left(g-\left(x-\Delta x\right)\right)}^{3}}}{m}}$$

Simplified Equation (8) is as follows:

(9) $$f_{e}=\frac{1}{2\pi }\sqrt{\frac{k-k_{S}-\frac{\varepsilon AV^{2}}{(g-x{)}^{3}{\left(1-\frac{-\Delta x}{g-x}\right)}^{3}}}{m}}=f_{0}\left(\sqrt{1-\beta -\frac{\gamma }{(1-\varphi {)}^{3}}}\right)\;$$

In Equation (8), $K_{e}^{\prime }$ is the corresponding electrostatic stiffness, and${\;f}_{0}$ is the intrinsic mechanical frequency:

(10) $$\gamma =\frac{K_{e}^{\prime }}{k},\;f_{0}=\frac{1}{2\pi }\sqrt{\frac{k}{m}},\;x=\frac{\frac{\varepsilon AV^{2}}{2g^{2}}}{k-\frac{\varepsilon AV^{2}}{g^{3}}}=\frac{k_{e}g}{2\left(k-k_{e}\right)},\;-\Delta x=\frac{m\cdot a}{k-k_{e}}\;$$

(11) $$K_{e}=\frac{\varepsilon AV^{2}}{g^{3}},\;{K_{e}}^{\prime }=\frac{\varepsilon AV^{2}}{{\left(g-x\right)}^{3}}=\frac{K_{e}\left(k-K_{e}\right)}{k-1.5K_{e}}\;,\;\beta =\frac{k_{S}}{k}\;$$

(12) $$\varphi =\frac{-\Delta x}{g-x}=\frac{2mga}{2kg^{2}-\frac{3\varepsilon AV^{2}}{g}}=\frac{2ma}{g\left(2k-3K_{e}\right)}$$

The following is further simplifying Equation (9):

(13) $$f_{e}=f_{0}\left(\sqrt{1-\beta -\gamma }-\frac{3}{2}\frac{\gamma }{\sqrt{1-\beta -\gamma }}\varphi +0(\varphi^{2})\right)$$

Acceleration sensitivity S is an important metric for evaluating accelerometer performance. The unit is Hz/m/s^2^. The following is according to its definition:

(14) $$|S|\approx \frac{\delta f}{\delta a}=f_{0}\left(\frac{-3\gamma }{\sqrt{1-\beta -\gamma }}\frac{m}{g\left(2k-3K_{e}\right)}\right)$$

Through finite element simulation, a CMUT array sensor designed for detecting acceleration is developed, with an acceleration measurement range of 0–20 g. Based on the CMUT structure, a schematic diagram of the parallel plate capacitor is illustrated in Figure 1. From top to bottom, it consists of an Al metal electrode, Si elastic membrane, cavity, SiO_2_ insulation layer, and Si substrate. As the structural unit is circular, the Si elastic membrane has a radius (R), thickness (h), and cavity height (g). The overall structure has a radius of R. The overall parameters of the CMUT structure are shown in Table 1, primarily detailing various parameters of the Si membrane material, such as structural dimensions, Young’s modulus (E), Poisson’s ratio (ν), and material density (ρ).

In this section, an analysis of the acceleration sensitivity of the traditional CMUT structure is conducted. Traditional CMUT structures typically feature a vacuum cavity. The CMUT accelerometer structure designed in this paper, however, incorporates an air cavity. As shown in Table 1, the membrane thickness h in the structure is 1 μm, and the acceleration sensitivity is 5.4 (Hz/g). To enhance the acceleration sensitivity of this sensor, the third section of this paper introduces a ring-perforation structure.

## 3. The CMUT Ring-Perforation Membrane Structure Study

To enhance the acceleration sensitivity of the CMUT accelerometer, this paper proposes a ring-perforation CMUT structure. The sensitivity of the CMUT sensor is related to the inherent structural stiffness. By reducing the mechanical stiffness k of the membrane, the acceleration sensitivity can be improved. The inherent mechanical stiffness of the CMUT vibrating membrane is given by the following:

$$k=\frac{192\pi D}{R^{2}}$$

where $D=\frac{Eh^{3}}{12(1-V^{2})}$. By altering the equivalent thickness of the upper flexible electrode, namely the elastic membrane, the structural stiffness k can be reduced, leading to an increase in acceleration sensitivity due to the decrease in mechanical stiffness.

**Figure 2:**
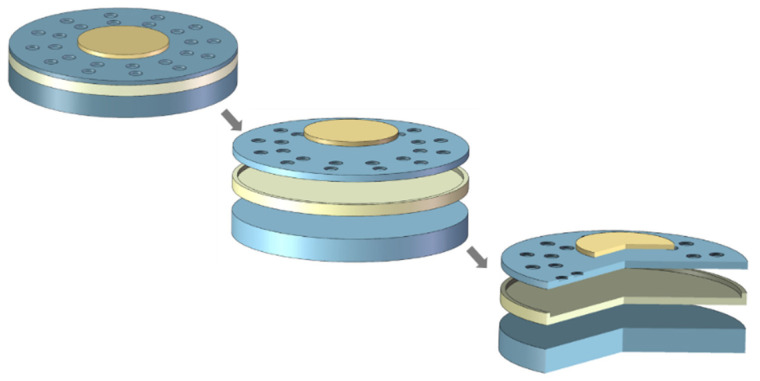
Three-dimensional structure of the CMUT cell ring-perforation membrane structure.

Through finite element simulation, the acceleration sensitivity of the structure is studied by establishing a ring-perforation structure, as shown in Figure 2. As depicted, initially, a traditional CMUT structure with a radius of 170 μm, membrane thickness h = 1 μm, and cavity height g = 0.2 μm is created. Subsequently, perforations are introduced into the membrane. In the traditional fabrication process, this step involves patterning through photolithography and subsequently employing deep silicon etching on the membrane surface. In the creation of the three-dimensional model presented in this paper, the circular perforations surround the center of the membrane in a 360° fashion and are evenly arranged in two circles on the membrane. In the finite element simulation, the electromechanical coupling physical field is applied, along with acceleration trostatic loads. In the solid mechanics module, the structure needs to be fixed around its perimeter. Finally, simulation calculations are performed for its characteristic frequencies.

To study the effect of the ring-perforation membrane structure on acceleration sensitivity, this paper considers the number of perforations, the radius of the perforations, and the position of the perforations from three aspects. Firstly, to ensure that all the structures can work properly in the simulation process, it is necessary to ensure that a reasonable voltage is selected. In the simulation of the ring-perforation membrane structure, 1.6 V is selected as the applied voltage. Under this, all the structures can work normally, and the membrane deflection does not exceed the maximum deflection. Considering the stability of the structure and the possibility of process realization, the thickness of the perforations is chosen to be 0.5 μm. Next, the number of perforations, the radius of the perforations, and the position of the perforations from three aspects were studied.

### 3.1. Analysis of the Effect of the Number of Perforations on Acceleration Sensitivity

**Figure 3:**
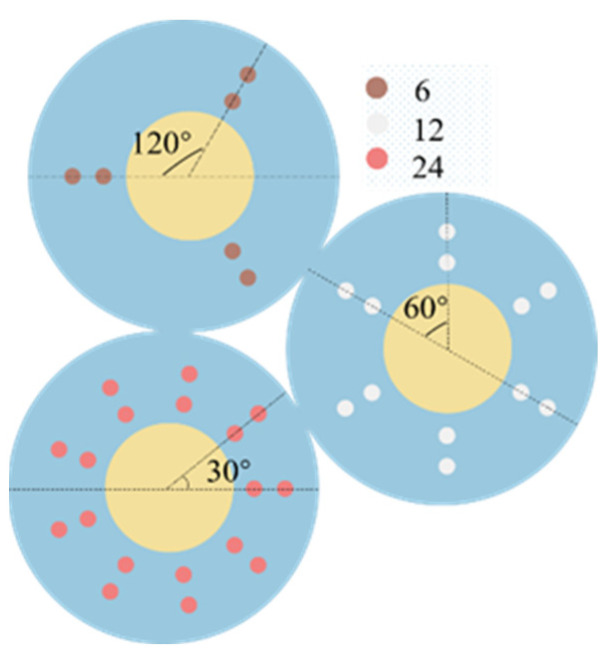
Two-dimensional diagrams of perforated structures with 6, 12, and 24 perforations.

In this section, the study investigates the influence of the number of perforations on the acceleration sensitivity of the structure. The perforations are uniformly distributed in a circular pattern around the center of the unit. For structures with different numbers of perforations, the angular spacing between circular perforations varies. In a single structural model, the angular spacing between circular perforations is kept constant. Figure 3 illustrates schematic diagrams of two-dimensional structures with different numbers of perforations, specifically 6, 12, and 24.

**Figure 4:**
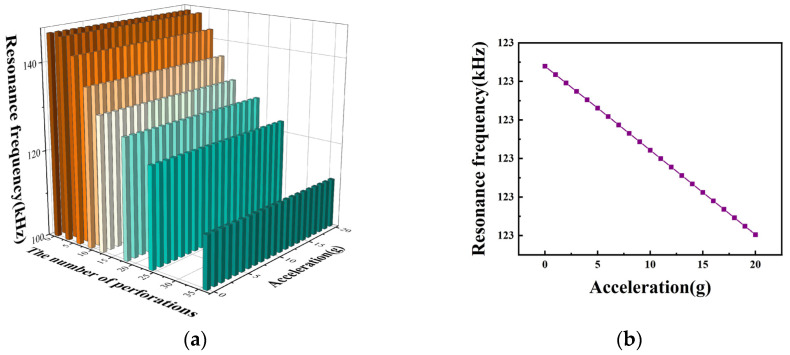
(**a**) Resonant frequency versus acceleration curves for the number of perforations 0, 3, 6, 9, 12, 18, 24, and 36; (**b**) resonant frequency variation curve with acceleration for the structure with 24 perforations.

In this study, it is necessary to ensure that the working voltage is certain to prevent the structure from being damaged due to excessive voltage, and 1.6 V is taken as the applied voltage. In addition to this, the effect of the number of perforations on the acceleration sensitivity of the structure needs to be ensured that the radius of the perforations, as well as the position of the perforations, are certain. In this study, the radius of the perforations is taken as 7 μm, and the position of the C_2_ perforations is taken as 70 μm for d_1_ and 30 μm for d_2_. After determining the voltage, radius of the perforations, and the position of the perforations, the study is carried out for the structures with the number of perforations of 0, 3, 6, 9, 12, 18, 24, and 36. The resonant frequency versus acceleration curves are shown in Figure 4—a resonant frequency variation curve with acceleration for the structure with 24 perforations. It can be seen from the figure that the resonant frequency of the device still varies linearly with acceleration at different numbers of perforations, and the linearity is well maintained.

**Figure 5:**
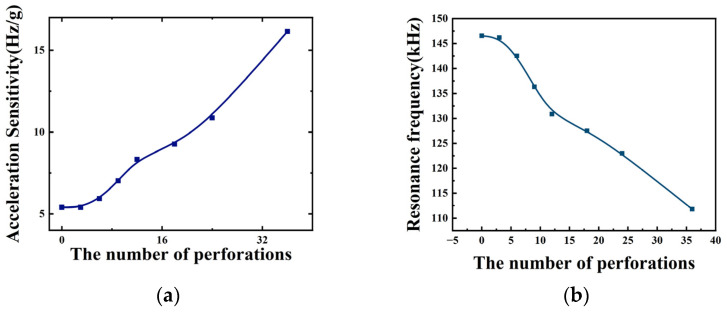
(**a**) Variation curve of acceleration sensitivity with the number of perforations; (**b**) variation curve of resonance frequency with the number of perforations for acceleration of 0.

As shown in Figure 5, the acceleration sensitivity of the device varies with the number of perforations, and the resonant frequency varies with the number of perforations when the acceleration is zero. From Figure 5a and Table 2, it can be seen that the acceleration sensitivity increases with the increase in the number of perforations. During the increase in the number of perforations from 0 to 36, the acceleration sensitivity increases from 5.4 (Hz/g) to 16.1 (Hz/g), which is a 200% increase in acceleration sensitivity. As shown in Figure 5b, as the number of perforations increases, the resonance frequency decreases when the acceleration is zero. As shown in Table 2, the resonant frequency decreases from 146.6 (kHz) to 111.8 (kHz). It can be concluded that as the number of perforations increases, the structure acceleration sensitivity also increases. Therefore, when selecting the number of perforations, we try to select a structure with a larger number of perforations. However, considering the problems of process realization and structural stability, we need to make a compromise here. In this paper, we chose a structure with 24 perforations.

### 3.2. Analysis of the Effect of the Perforation Radius on Acceleration Sensitivity

**Figure 6:**
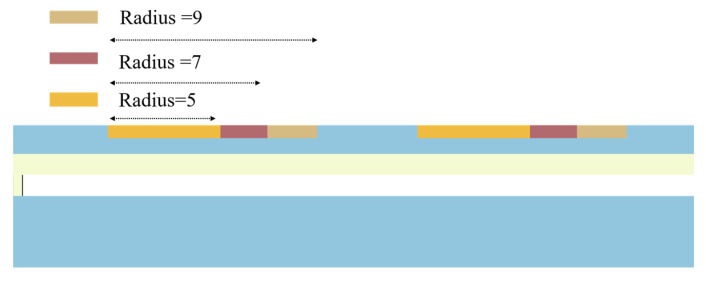
Two-dimensional diagrams of perforated structures with perforation radii of 5 μm, 7 μm, and 9 μm.

In this subsection, the effect of perforation radius on the acceleration sensitivity of the device is investigated and analyzed at an applied voltage of 1.6 V, several perforations of 24, and a C_2_ perforation position of 30 μm. As shown in Figure 6, it is a schematic diagram of half of the CMUT structure. The study examines partial perforation radii of 5 μm, 7 μm, and 9 μm. As depicted, when the circular perforations are formed, the position of the perforations remains constant. During the increase in perforation radius, the equivalent thickness of the membrane decreases.

**Figure 7:**
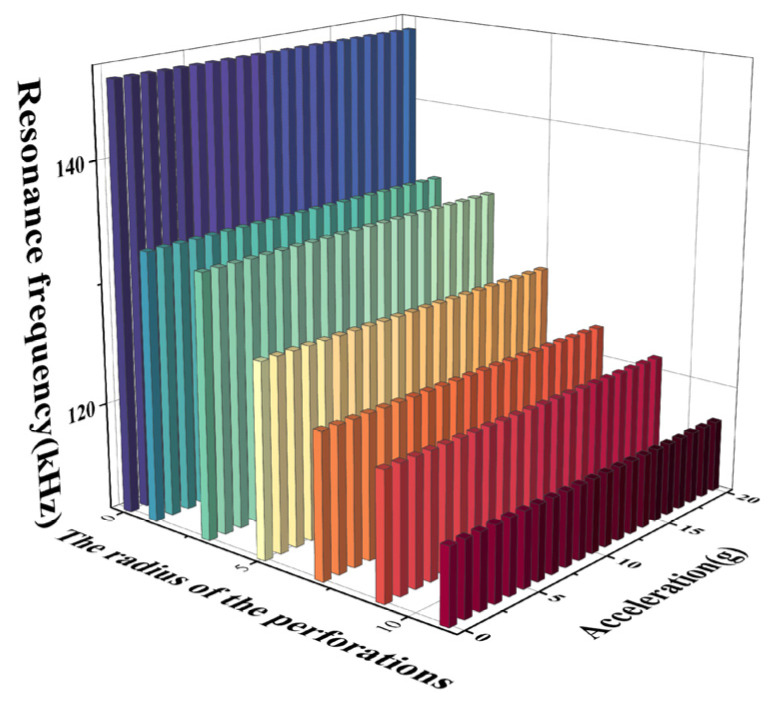
Curve of resonant frequency versus acceleration for perforation radius 0–11 μm.

The resonance frequency trend is studied by applying an acceleration load and an electrostatic load to the sensor structure with perforation radii of 0 μm, 1 μm, 3 μm, 5 μm, 7 μm, 9 μm, and 11 μm, respectively. Figure 7 shows the resonance frequency variation curve with acceleration at different perforation radii, from which it can be seen that the resonance frequency decreases with the increase in acceleration. Under different perforation radii, the linearity of the structural frequency response curve remains good.

**Figure 8:**
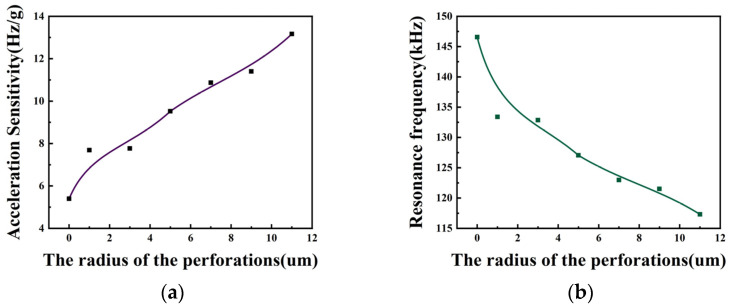
(**a**) Curve of acceleration sensitivity versus perforation radius; (**b**) curve of resonance frequency versus perforation radius for acceleration of 0.

From Figure 8a and Table 3, it can be seen that increasing the perforation radius causes an increase in the acceleration sensitivity. As the perforation radius increases from 0 μm to 11 μm, the acceleration sensitivity increases from 5.4 (Hz/g) to 13.2 (Hz/g). From Figure 8b and Table 3, it can be seen that as the perforation radius increases from 0 μm to 9 μm, the resonance frequency of the device decreases from 146.6 kHz to 117.3 kHz at acceleration 0. It is concluded from the analysis that the increase in the perforation radius is effective in increasing the acceleration sensitivity of the structure. In order to ensure sensitivity and structural stability at the same time, the structure with the 7 μm perforation radius is selected as the size of the acceleration measurement in this paper.

### 3.3. Analysis of the Effect of C_2_ Perforation Position on Acceleration Sensitivity

**Figure 9:**
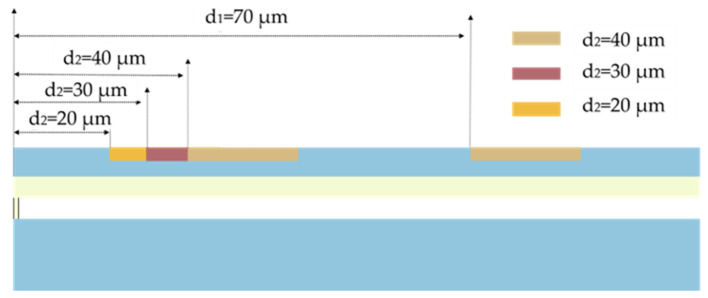
Schematic diagrams of two-dimensional structures with C_2_ perforation positions (d_2_) of 20 μm, 30 μm, and 40 μm.

In this section, the effect of the perforation position on the acceleration sensitivity of the device is investigated. In the studied ring-perforation structure, perforations need to be positioned away from the electrode locations.For device performance assurance, an electrode radius of 80 μm is selected in this paper. Simultaneously, to ensure the stability of the ring-perforation structure, in the model, the perforation position C_1_ is kept as far away from the electrode as possible. Figure 9 illustrates the C_2_ perforation positions, denoted as d_2_, are 20 μm, 30 μm, and 40 μm, respectively, in schematic diagrams of two-dimensional structures.

**Figure 10:**
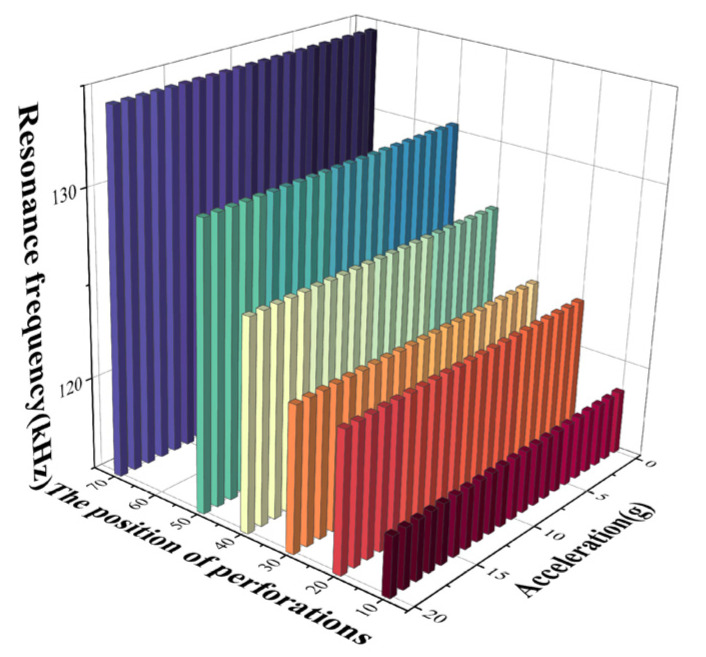
Resonance frequency versus acceleration curves for different perforation positions.

In the actual modeling process, with the opening radius and number determined, frequency analysis of the device is conducted by varying the position of C_2_ (denoted as d_2_) while keeping the C_1_ perforation position (d_1_) fixed at 70 μm. The analysis is carried out at an applied voltage of 1.6 V, with a number of perforations of 24 and a radius of perforations of 7 μm. An acceleration load and an electrostatic load are applied to the structure at C_2_ position d_2_ taken as 10 μm, 20 μm, 30 μm, 40 μm, 50 μm, and 70 μm to study the trend of resonance frequency variation. From Figure 10, it is known that the resonant frequency decreases with the increase in acceleration at different perforation positions. Additionally, the linearity of the frequency response curve of the structure is well maintained under different perforation positions.

**Figure 11:**
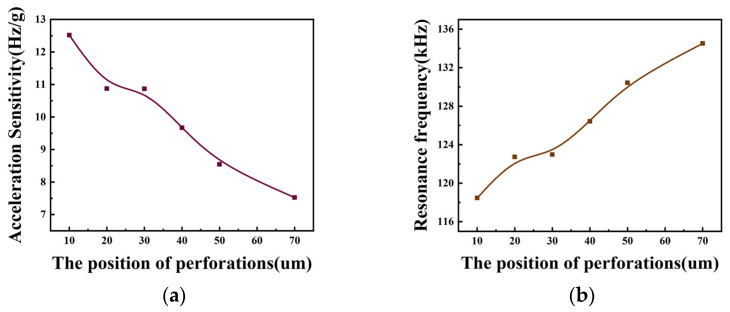
(**a**) Variation curve of acceleration sensitivity with perforation position; (**b**) variation curve of resonance frequency with perforation position at the acceleration of 0.

From Figure 11a, it can be seen that the acceleration sensitivity decreases from 12.5 (Hz/g) to 7.5 (Hz/g) as the C_2_ position d_2_ is increased from 10 μm to 70 μm. The acceleration sensitivity when d_2_ is taken as 10 μm is increased by 50% compared to when d_2_ is 50 μm. Compared to the acceleration sensitivity at d_2_ of 70 μm, the acceleration sensitivity increases by 70% at d_2_ of 10 μm and 13% at d_2_ of 50 μm. Meanwhile, from Figure 8b, it can be seen that as C_2_ is closer to the edge of the membrane, the resonant frequency of the device is smaller. As shown in Table 4, in order to ensure the stability of the structure, this paper selects the C_2_ position of 30 μm for the study. Meanwhile, from Figure 11b, it can be seen that C_2_ is closer to the edge of the membrane. The resonant frequency of the device is smaller. As shown in Table 4, in order to ensure the stability of the structure, this paper selects the C_2_ position of 30 μm for the study.

### 3.4. Analysis of the Effect of Voltage on Acceleration Sensitivity

**Figure 12:**
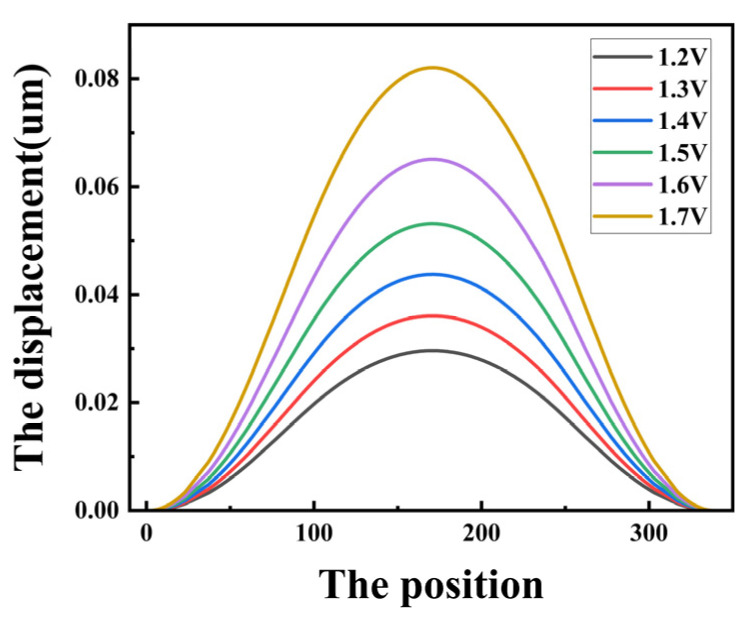
Maximum membrane displacement at different voltages.

To determine the applied voltage of the CMUT cell, based on the small deflection assumption as well as the classical Kirchhoff theory, it should be integrated into the design of the sensor that the maximum displacement of the thin plate should be less than 20% of its thickness. The expression for the operating voltage of the parallel plate capacitor is $V=\sqrt{\frac{8Kg^{3}}{27\varepsilon A}}$ where *K* is the inherent elastic stiffness of the parallel plate capacitor, *g* is the gap between the plates, *A* is the area between the plates, and *ε* is the dielectric constant. When the elastic stiffness *K* decreases, the operating voltage of the parallel plate capacitor will also decrease. In order to prevent the sheet from collapsing during operation and causing damage to the device, the flexible sheet displacement should also be less than 45% of the spacing between the upper and lower pole plates. In this section, a CMUT cell structure with several perforations of 24, a perforation radius of 7 μm, and a C_2_ perforation position d_2_ of 30 μm are simulated. The longitudinal displacement of the membrane is calculated for different voltages with only electrostatic force applied. As shown in Figure 12, the membrane displacement increases as the voltage increases. In this case, the membrane displacement of the device at a voltage of 1.6 V is 0.065 μm, while the device perforation height is 0.2 μm, and the device has not reached the maximum displacement during collapse at this voltage.

**Figure 13:**
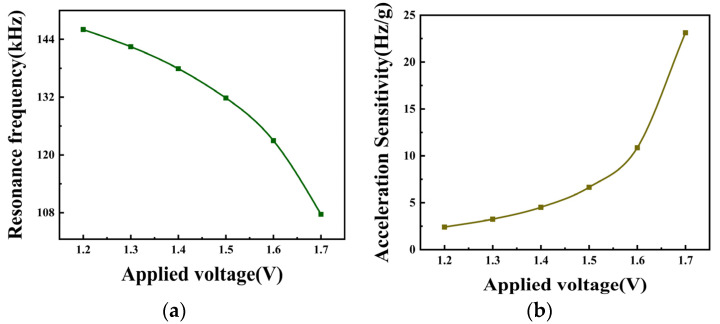
(**a**) CMUT cell resonant frequency versus voltage curve; (**b**) acceleration sensitivity versus voltage curve.

In order to select the appropriate applied voltage, the device sensitivity under different electrostatic loads needs to be investigated. In this regard, the acceleration sensitivity of the device at 1.2 V, 1.3 V, 1.4 V, 1.5 V, 1.6 V, and 1.7 V is simulated. As shown in Figure 13a, the resonant frequency varies with voltage for an acceleration of zero. As the voltage increases, the resonant frequency decreases, and when the voltage increases from 1.2 V to 1.7 V, the resonant frequency also decreases from 146.0 kHz to 107.7 kHz. The acceleration sensitivity versus operating voltage is shown in Figure 13b for acceleration of 0–20 g. From Table 5, it can be seen that when the voltage is increased from 1.2 V to 1.7 V, the acceleration sensitivity is increased from 2.4 (Hz/g) to 23.1 (Hz/g). When we consider the sensitivity, device damage, and other factors in the actual design stage, this paper selects 1.6 V as the device’s applied voltage.

**Figure 14:**
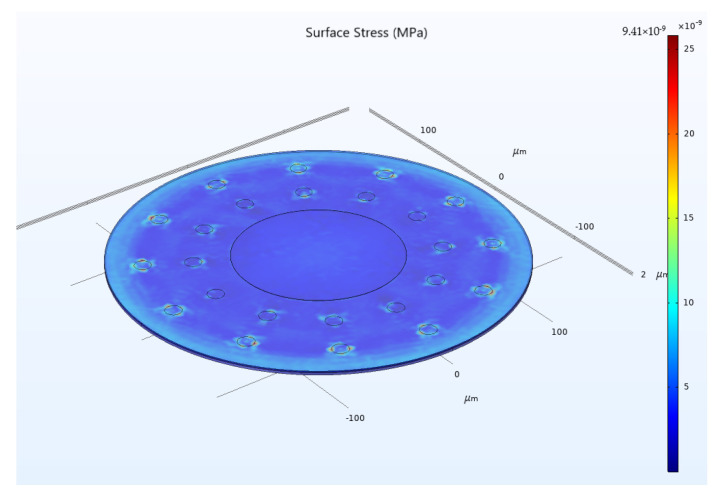
Maximum stress on the membrane of the CMUT cell structure at a voltage of 1.6 V and an acceleration of 20 g.

During the operation of CMUT devices, the elastic membrane undergoes continuous vibration. The ring-perforation structure designed in this paper may lead to excessive membrane stress during operation, resulting in structural instability. In order to simultaneously ensure the acceleration sensitivity and structural stability of the ring-perforation membrane structure, the stresses of the CMUT cell when both an electrostatic load and a 20 g acceleration load are simultaneously applied need to be analyzed. As shown in Figure 14, the maximum stress is 9.41 × 10^−9^ MPa, which is much smaller than the yield strength of silicon, 7000 MPa. Therefore, both the applied voltage and the structural design are reasonable.

## 4. Contrast and Discussion

The conventional CMUT structure has low sensitivity in acceleration measurement, and the ring-perforation membrane structure designed in this paper can effectively improve acceleration sensitivity. This paper investigates the effects of the number of perforations, perforation radius, and perforation position on the acceleration sensitivity of the CMUT membrane. From Table 6, it can be seen that the acceleration sensitivity is improved by increasing the number of perforations compared to the number of perforations at zero. When the number of perforations is six, the acceleration sensitivity increases by 10%. When the number of perforations is increased to 36, the acceleration sensitivity increases by 200%. When only the perforation radius was changed, the acceleration sensitivity was 40% at a perforation radius of 1 μm, and the acceleration sensitivity was improved by 140% at a perforation radius of 11 μm, compared with that when the number of activities was zero. When examining the effect of perforation position on acceleration, the acceleration sensitivity increases as the C_2_ perforation position moves closer to the membrane edge when only the C_2_ perforation position d_2_ is changed. A 130% increase in acceleration sensitivity is achieved at 10 μm for the d_2_ position, and a 40% increase in acceleration sensitivity is achieved at 70 μm for the d_2_ position. The CMUT ring-perforation membrane structure increases the acceleration sensitivity by 100% compared with the traditional structure of CMUT. In the study of the relationship between voltage and acceleration sensitivity, as the voltage increases, the acceleration sensitivity will be higher, and the elastic membrane displacement will be larger.

## 5. Summary and Prospects

This paper presents a study of CMUT acceleration sensors and designs a CMUT ring-perforation membrane structure. The relationship between the acceleration and the resonance frequency of the CMUT cell is established by combining the electromechanical coupling model of the CMUT. Through simulation, it is concluded that the acceleration sensitivity of the traditional CMUT structure is low, and the acceleration sensitivity in the range of 0–20 g is 5.4 Hz/g. The acceleration sensitivity of the ring-perforation membrane structure proposed in this paper can reach 10.9 Hz/g, an increase of 100%. In the study of the ring-perforation membrane structure, the number of perforations was increased from 6 to 36, and the acceleration sensitivity was increased by 170%. The perforation radius is increased from 1 μm to 11 μm, and the acceleration sensitivity is improved by 70%. C_2_ perforation position d_2_ from 70 μm to 10 μm, and acceleration sensitivity increased by 70%. An increase in the number of perforations and perforation radius causes an increase in acceleration sensitivity, and the closer the C_2_ perforation location is to the edge of the membrane, the higher the acceleration sensitivity. In this paper, the feasibility of acceleration measurement based on CMUT arrayed sensors is verified by simulation. Due to their excellent resonance characteristics, better stability, and array integration, CMUT sensors have great advantages in future miniaturization and multi-functionalization, with more and more in-depth research on CMUT sensors.

## Figures and Tables

**Table 1:** Material properties and structural parameters of Si elastic membrane.

Parameter	Value
Radius R	170 µm
Thickness h	1 µm
Poisson’s ratio v	0.29
Separation distance g	0.2 µm
Young’s modulus E	169 GPa
Density ρ	2.332 kg/m^3^
Acceleration Sensitivity	5.4 (Hz/g)

**Table 2:** Performance parameters of the ring-perforation membrane structure with different numbers of perforations.

Numbers of Perforations	Resonance Frequency at Acceleration 0 (kHz)	Sensitivity (Hz/g)
0	146.6	5.4
3	146.2	5.4
6	142.5	5.9
9	136.3	7.0
12	130.8	8.3
18	127.5	9.3
24	123	10.9
36	111.8	16.1

**Table 3:** Performance parameters of ring-perforation membrane structure with different perforation radii.

The Radius of Perforation (μm)	Resonance Frequency at Acceleration 0 (kHz)	Sensitivity (Hz/g)
0	146.6	5.4
1	133.4	7.7
3	132.8	7.8
5	127.1	9.5
7	123.0	10.9
9	121.5	11.4
11	117.3	13.2

**Table 4:** Performance parameters of the ring-perforation membrane structure with different C_2_ positions.

Perforation Position d_2_ (μm)	Resonance Frequency at Acceleration 0 (kHz)	Sensitivity (Hz/g)
70	134.5	7.5
50	130.4	8.5
40	126.4	9.7
30	123.0	10.9
20	122.7	10.9
10	118.5	12.5

**Table 5:** Performance parameters of the ring-perforation membrane structure at different voltages.

Voltage (V)	Displacement (μm)	Resonance Frequency at Acceleration 0 (kHz)	Sensitivity (Hz/g)
1.2	0.03	146	2.4
1.3	0.036	142.5	3.2
1.4	0.044	137.9	4.5
1.5	0.053	131.8	6.6
1.6	0.065	123.0	10.9
1.7	0.082	107.7	23.1

**Table 6:** Comparison of acceleration sensitivity of the ring-perforation membrane structure.

Number	Radius	d_2_	Resonance Frequency at Acceleration 0 (kHz)	Sensitivity (Hz/g)	Percentage Upgrade
0	0	0	146.6	5.4	
6	7	30	142.5	5.9	9.30%
36	7	30	111.8	16.1	198.10%
24	1	30	133.4	7.7	42.60%
24	11	30	117.3	13.2	144.40%
24	7	10	118.5	12.5	131.50%
24	7	70	134.5	7.5	38.90%
24	7	30	123.0	10.9	101.90%

## Data Availability

Data are contained within the article.
